# Multi-domain feature joint optimization based on multi-view learning for improving the EEG decoding

**DOI:** 10.3389/fnhum.2023.1292428

**Published:** 2023-12-07

**Authors:** Bin Shi, Zan Yue, Shuai Yin, Junyang Zhao, Jing Wang

**Affiliations:** ^1^Xi’an Research Institute of High-Technology, Xi’an, Shaanxi, China; ^2^Institute of Robotics and Intelligent System, School of Mechanical Engineering, Xi’an Jiaotong University, Xi’an, China

**Keywords:** brain-computer interface, common spatial pattern, electroencephalogram, motor imagery, multi-domain feature joint optimization

## Abstract

**Background:**

Brain-computer interface (BCI) systems based on motor imagery (MI) have been widely used in neurorehabilitation. Feature extraction applied by the common spatial pattern (CSP) is very popular in MI classification. The effectiveness of CSP is highly affected by the frequency band and time window of electroencephalogram (EEG) segments and channels selected.

**Objective:**

In this study, the multi-domain feature joint optimization (MDFJO) based on the multi-view learning method is proposed, which aims to select the discriminative features enhancing the classification performance.

**Method:**

The channel patterns are divided using the Fisher discriminant criterion (FDC). Furthermore, the raw EEG is intercepted for multiple sub-bands and time interval signals. The high-dimensional features are constructed by extracting features from CSP on each EEG segment. Specifically, the multi-view learning method is used to select the optimal features, and the proposed feature sparsification strategy on the time level is proposed to further refine the optimal features.

**Results:**

Two public EEG datasets are employed to validate the proposed MDFJO method. The average classification accuracy of the MDFJO in Data 1 and Data 2 is 88.29 and 87.21%, respectively. The classification result of MDFJO was significantly better than MSO (*p* < 0.05), FBCSP_32_ (*p* < 0.01), and other competing methods (*p* < 0.001).

**Conclusion:**

Compared with the CSP, sparse filter band common spatial pattern (SFBCSP), and filter bank common spatial pattern (FBCSP) methods with channel numbers 16, 32 and all channels as well as MSO, the MDFJO significantly improves the test accuracy. The feature sparsification strategy proposed in this article can effectively enhance classification accuracy. The proposed method could improve the practicability and effectiveness of the BCI system.

## 1 Introduction

Brain–computer interface (BCI) technology realizes direct communication and control between the brain and electronic devices based on cortex electrical signals. By not relying on conventional brain output pathways, BCI opens up entirely new ways for the human brain to communicate and control information with the outside world (Maslova et al., 2023). There are many patterns of brain electrical signals in BCI: electroencephalogram (EEG), electrocorticography (ECoG), functional magnetic resonance imaging (fMRI), and positron emission tomography (PET) (Sharma et al., 2023). The EEG is extensively applied to collect brain signals in BCI systems since it is inexpensive, portable, and non-invasive, and has relatively high temporal resolution (McFarland and Wolpaw, 2017; Padfield et al., 2019). Common EEG-BCI systems include steady-state visual evoked potential (SSVEP), event-related P300, N400, motor imagery (MI), and slow cortical potential.

Over the past 2 decades, many researchers have focused on the research of BCI based on motor imagery (MI) (Pfurtscheller and Neuper, 2001; Chepurova et al., 2022) and have confirmed its application as neurorehabilitation (Brusini et al., 2021; Choy et al., 2023), neuroprosthetics (Neuper et al., 2006), and gaming (Laar et al., 2010). Motor imagery might be seen as a mental rehearsal of a motor act without any overt motor output and could activate certain brain regions (Pfurtscheller and Neuper, 2001). Sensory stimulation, motor behavior, and mental imagery could change the functional connectivity within the cortex and result in an amplitude suppression [event-related desynchronization (ERD)] or in an amplitude enhancement [event-related synchronization (ERS)] of mu and beta rhythms. Mu rhythm is in the range of 7–13 Hz, and the beta rhythm is in the range of 13–30 Hz, both originating in the sensorimotor cortex (Blankertz, 2008).

The classical EEG-BCI system mainly consists of signal acquisition, signal processing, classification recognition, and feedback/application. The signal processing includes signal preprocessing, feature extraction, and feature selection. The main purpose of signal preprocessing is to remove artifacts. Feature extraction means extracting features from clean EEG signals and common extraction methods include discrete wavelet transform (DWT) (Zhou et al., 2018), empirical mode decomposition (EMD) (Mohamed et al., 2018), power spectral density (PSD) (Rodríguez-Bermúdez and García-Laencina, 2012), Hilbert transform (Zhou et al., 2018), and common spatial pattern (CSP). Feature selection could eliminate irrelevant or redundant features so as to reduce the number of features, improve model accuracy, and reduce running time.

The basic principle of the CSP algorithm is to use the diagonalization of the matrix to find a set of optimal spatial filters for projection so as to maximize the difference between the variance values of the two types of signals and obtain a feature vector with a high degree of differentiation (Blankertz et al., 2007; Benjamin Blankertz et al., 2008; Blankertz, 2008; Vidaurre et al., 2009). The CSP is widely used to extract features (Blankertz et al., 2007; Li et al., 2011; Tangermann et al., 2012; Baig et al., 2017; Li et al., 2017). For the MI-BCI system, the effectiveness of CSP is highly affected by the frequency band and time window of EEG segments and channels selected (Blankertz, 2008; Miao et al., 2017a,b).

In general, before feature extraction using CSP, EEG signals are filtered within a fixed broad frequency band, e.g., 8–30 Hz (Jin et al., 2019; Jin J. et al., 2020) and 4–40 Hz (Zhang et al., 2015, 2021; Jiao et al., 2019, 2020). However, given the intrasubject variability in the frequency band of reactive components (Pfurtscheller et al., 2006), selecting subject-specific optimal frequency bands contributes to the extraction of discriminative features. The existing studies have confirmed that variants of CSP [SBCSP (Quadrianto Novi et al., 2007), FBCSP (Ang et al., 2008), DFBCSP (Thomas et al., 2009), and SFBCSP (Zhang et al., 2015)] could improve the classification rate of MI by optimizing the optimal frequency band using mathematical statistics.

Most existing studies utilize a fixed time segment to extract features by CSP, which results in suboptimal feature extraction since the time interval when the brain responses to the mental tasks occur may not be accurately detected. Therefore, an appropriate time window of EEG should be preselected to cover the interval when the EEG pattern is activated and remove those unrelated sampling points. The correlation-based time window selection (CTWS) was developed for MI-based BCIs (Feng et al., 2018). The two Parzen window-based method was proposed to select the discriminative feature subset and subject-specific time segment (Wang et al., 2020). Furthermore, the effectiveness of CSP is highly affected by the frequency band and time interval of EEG segments.

The frequency band and time interval selection mainly include heuristic ways and the mathematical optimization method. On the one hand, some studies use the heuristic method to optimize features in multiple time windows and bandwidths (Ang et al., 2012; Zhang et al., 2019; Miao et al., 2021; Yuan et al., 2021). The main purpose is to optimize the selection of the frequency band and time interval and then carry out feature extraction. On the other hand, the main idea of the mathematical optimization method is to divide time intervals and frequency bands, obtaining multiple sub-bands and time segments. Then, the high-dimensional feature sets are constructed by extracting features on sub-bands and time segments through the CSP algorithm and selecting features through mathematical optimization or statistical methods. Most commonly, the time-frequency feature selection by LASSO includes TSGSP (Zhang et al., 2019), CTFSP (Miao et al., 2021), and mutual information (Ang et al., 2012).

Apart from frequency band and time window optimization, another important issue to consider is the determination of an appropriate EEG channel combination for the spatial pattern. Channel selection can improve performance and user comfort while reducing the cost of the system (Xu et al., 2021). Recently, numerous channel selection methods, working toward either selecting the most effective channels or eliminating noisy channels, have been proposed for motor imagery EEG applications (Jin et al., 2019; Jin J. et al., 2020; Qi et al., 2021; Faye and Islam, 2022).

The above-mentioned methods are targeted at frequency band, time interval, time-frequency feature optimization, and channel selection to improve the performance of MI-BCI. Furthermore, there are also studies to optimize the features of the spatial-frequency domain or time-frequency-spatial domain. Multi-view learning aims to improve the learning performance of target tasks by using the relationship or mutual learning between view data (Xu et al., 2013). According to different perspectives of specific learning tasks, it can be divided into the multi-view classification method (Yu et al., 2014; Bekker et al., 2016), multi-view clustering method (Zhao et al., 2014; Wang et al., 2016), and multi-view feature selection/dimensionality reduction method (Yuan et al., 2021; Qiang et al., 2022). The multi-scale optimization (MSO) method was proposed by introducing multi-view feature selection to optimize filter bands over multiple channel sets within CSPs (Jiao et al., 2020). Moreover, a novel framework termed the time window filter bank common spatial pattern with multi-view optimization was proposed (Huang et al., 2021).

Likewise, some studies have been mainly centered around investigating either frequency band and time window selection or spatial-frequency optimization. Few studies focus on joint optimization of time-frequency-spatial features. In this study, a novel framework termed multi-domain feature joint optimization (MDFJO) based on multi-view learning is proposed to select the discriminative features. Our contributions are summarized as follows:

1. We investigated the joint optimization of the filter bands and time intervals over multiple channel sets within CSPs by multi-view learning.
2. On the basis of selecting features, the feature sparsification strategy was studied to reduce the feature dimensionality.
3. Two public motor imagery EEG databases were used, and the performance of the proposed method was compared with existing methods to verify its effectiveness.

The rest of the article is organized as follows. The proposed MDFJO method is illustrated in the Method section. The experimental results are described in detail in the Results section. In the Discussion section, we analyzed the parameters and discussed the potential extensions of our method for future studies. Finally, a summary of this study is given in Section 5.

## 2 Method

In this part, EEG data, channel selection, and feature extraction methods are described, followed by a detailed presentation of the proposed method MDFJO. Furthermore, the parameters of the proposed method and the comparison method are selected. First, two EEG public datasets for validation of the method are described below.

### 2.1 EEG Data

**Figure 1:**
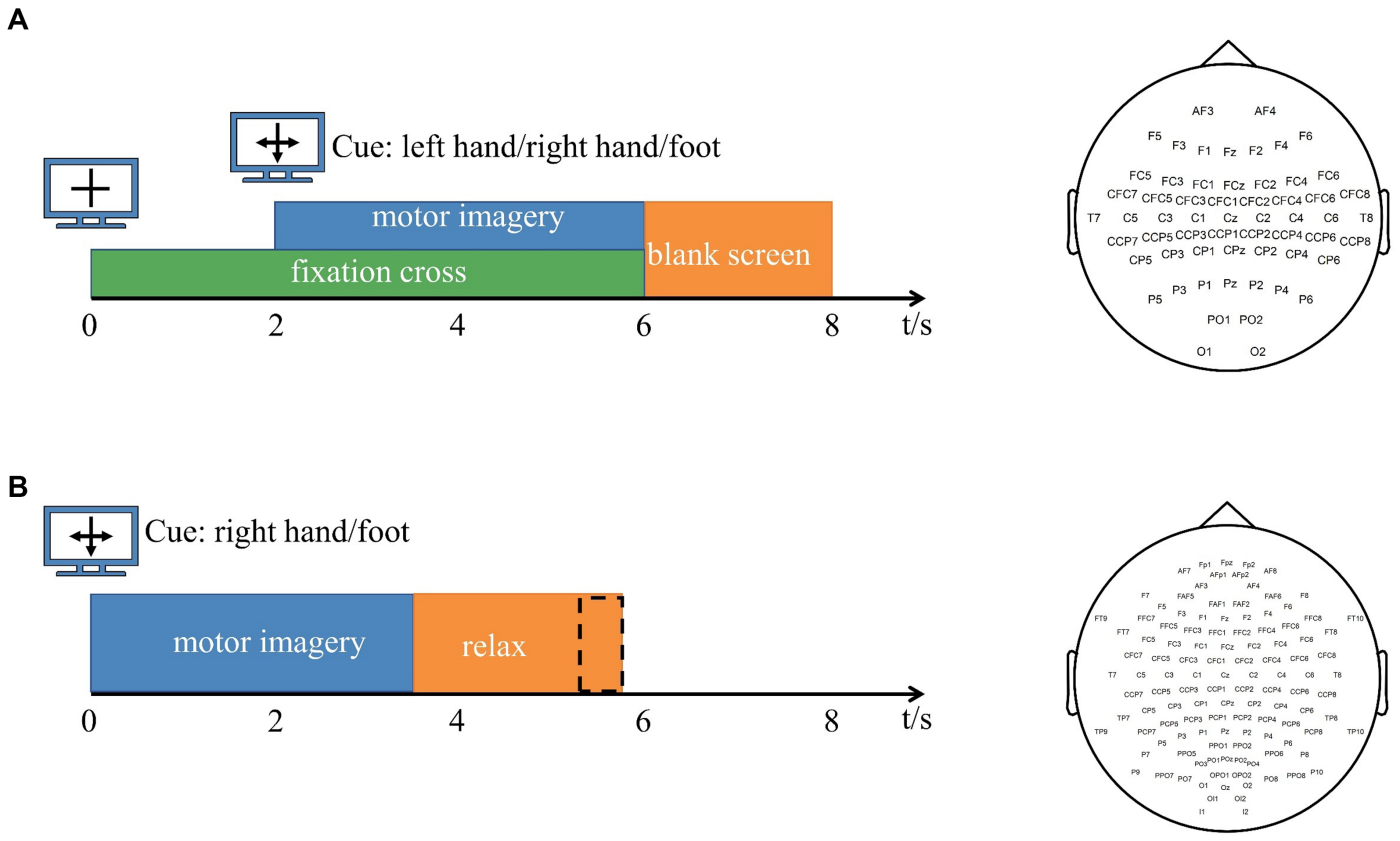
**(A)** Experimental paradigm and channel arrangement for BCI Competition IV dataset 1. Arrows pointing left, right, or down have been presented as cues for imagining left hand, right hand, or foot movements. After a fixation cross was presented for 2 s, the directional cue was overlaid for 4 s. Then, the screen was blank for 2 s. The number of channels is 59. **(B)** Experimental paradigm and channel arrangement for BCI Competition III dataset IVa. Within 3.5 s of the visual cues display, the subjects performed the right hand or right foot motor imagery according to the cue. The presentation of target cues is intermitted by periods of random length, 1.75 to 2.25 s, in which the subject could relax. The number of channels is 118. The channel arrangement of Data 1 and Data 2 follows the 10–20 international standard lead system.

1. Data 1: The dataset is derived from BCI Competition IV dataset 1 (Tangermann et al., 2012). The EEG signals of seven participants (“a,” “b,” “c,” “d,” “e,” “f,” and “g”) were recorded, and the number of channels was 59. The data of each participant included calibration data for 200 trials and test data for 200 trials, and the calibration data were used in this study. In each trial, each participant performed a pre-set motor imagery task (right hand and left hand or foot) for 4 s. For subjects “a” and “f,” the motor imagery task involved the left hand and foot. Other subjects performed left- and right-handed motor imagery tasks. The sampling frequency is 100 Hz. The timing scheme of the paradigm and channel arrangement are shown in Figure 1A.
2. Data 2: BCI Competition III dataset IVa (Blankertz et al., 2006) was used for experimental method validation in this study. The EEG signals of five subjects (“aa,” “al,” “av,” “aw,” and “ay”) were included, and the number of channels was 118. The raw data are downsampled to 100 Hz. Each participant performed 280 trials. In each trial, each subject performed a pre-set motor imagery task (right hand and right foot) for 3.5 s. The timing scheme of the paradigm and channel arrangement are shown in Figure 1B. Both channel arrangement conforms to the 10–20 international standard lead system.

### 2.2 Channel selection and feature extraction

The continuous EEG data for each dataset are segmented into single-trial data, and then common average reference (CAR) is applied for the spatial filter to enhance the signal-to-noise ratio (McFarland et al., 1997). Furthermore, a fifth-order Butterworth band-pass filter (4–40 Hz) is used for filtering the EEG signal (Jiao et al., 2019, 2020). Channel selection aims to select the channel combination with the most feature difference for a specific subject to obtain better classification performance. The FDC is regarded as the channel selection method. The discriminative power of each channel is calculated by the FDC value between the two classes. First of all, time segmentation is conducted by using rectangular time windows (100 points) and the length of signal (250 points) for Data 1 (100 Hz × 4 s) and Dataset IVa (100 Hz × 3.5 s), respectively. The 50% overlapping is used in neighboring *t*-segments for two datasets. $P_{ch,t}=\log\left(\mathrm{var}\left(x_{ch,t}\right)\right)$ is calculated as the feature of each segment, where $x_{ch,t}$ is signal data of the *t*-segment for channel *ch*. $P_{ch,t}$ denotes log-power. Then, the FDC value between two classes is $\phi_{ch,t}={\left(m_{1}-m_{2}\right)}^{2}/\left(\mathrm{var}\left(P_{ch,t}^{1}\right)+\mathrm{var}\left(P_{ch,t}^{2}\right)\right)$ , where *m*_1_ and *m*_2_ are means of $P_{ch,t}$ of all trials in two classes, $P_{ch,t}^{1}$ and $P_{ch,t}^{2}$ denote log-power of two classes, respectively. Finally, the maximum FDC of all *t*-segments is taken as the FDC value of each channel. The FDC values of all channels are arranged in descending order, and the channels corresponding to the first FDC values are selected in this study.

The common spatial pattern (CSP) is an efficient feature extraction algorithm that has been widely utilized in MI-based BCI systems. CSP is realized by the simultaneous diagonalization of two classes of signal-covariance matrices. After removing the mean value of the preprocessing data, the single-trial EEG data were represented as a matrix $X_{d}\in R^{M\times T}$ , where M is the number of channels and T is the time point for each channel. $X_{d},d\in \left\{1,2\right\}$ represents the EEG signal of class *d*. CSP seeks projection vectors by maximizing the ratio of the transformed data variance between two classes. The optimal spatial filters $W=\left[w_{1},\ldots ,w_{2m}\right]\in R^{M\times 2m}$ were formed with the first and last *m* projection vectors. Finally, the EEG data of each trial X were projected by W to obtain the new signal $Z=W^{T}X$ . In this study, *m* = 1. Feature vector $f_{p}$ is expressed as follows:


(1) $$f_{p}=\log\left(\mathrm{var}\left(Z_{p}\right)\right),p=1,\ldots ,2m$$

The two types of features were obtained by the CSP algorithm, and the features and corresponding labels were imported into the classification algorithm to train the classifier. A support vector machine (SVM) classification method with a radial basis function kernel is applied (Chang and Lin, 2007).

### 2.3 Multi-domain feature joint optimization

The proposed MDFJO method mainly includes a channel pattern division based on FDC, sub-band division, and time interval division, which is feature selection based on multi-view learning and feature sparsification strategy and MDFJO method implementation steps.

#### 2.3.1 Channel pattern division based on FDC

**Figure 2:**
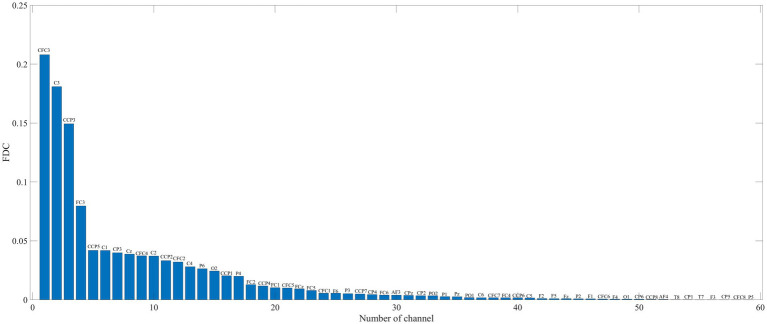
FDC value of subject b from Data 1. Channel labels are displayed on each bar.

The FDC was used to compute the channel weights and sort the channel in descending order. The subject b from Data 1 is taken as an example, and its FDC value is shown in Figure 2. All channels were decomposed into a three-channel mode. More specifically, the first 16 channels are selected in descending order as a channel combination mode, namely, channels CFC3 to CCP1. According to the above principle, we get a combination mode of 32 channels, namely, channels CFC3 to CP2. Furthermore, all channels are regarded as in a combination mode. In this study, there were three modes, s_1_ = 16 and s_2_ = 32. The third mode is to use all the channels, for Data 1, s_3_ = 59, and Data 2, s_3_ = 118.

#### 2.3.2 Sub-band and time interval division

The EEG signals in each channel mode are divided into time interval with a time window length of 2 s and an overlap time length of 0.5 s. For Data 1, each channel mode has five time intervals, namely, t_1_ = 0–2 s, t_2_ = 0.5–2.5 s, t_3_ = 1–3 s, t_4_ = 1.5–3.5 s, and t_T_ = 2–4 s. For Data 2, there are only four time intervals, namely, 0–2 s, 0.5–2.5 s, 1–3 s, and 1.5–3.5 s because the length of motor imagery time is 3.5 s. After that, the EEG signals in the 4 to 40 Hz frequency band in each time window were filtered by 4 Hz bandwidth and 2 Hz overlap frequency width. Thus, 17 filtering sub-bands are obtained. The frequency band of 4–40 Hz is divided, the bandwidth is 4 Hz, the overlap rate is 50%, that is, 4–8 Hz, 6–10 Hz, 8–12 Hz…, 36–40 Hz, totaling 17 sub-bands. Subsequently, the CSP features were extracted from each sub-band EEG signal.

#### 2.3.3 Feature selection based on multi-view learning

In the real world, an object is often described by multiple views. For example, an image has various heterogeneous features through different descriptors, such as RGB, LBP, HOG, and SURF. Different views represent different aspects of an object and can provide more information than a single view. In the past decades, according to different perspectives of specific learning tasks, it can be divided into multi-view classification method (Yu et al., 2014; Bekker et al., 2016), multi-view clustering method (Zhao et al., 2014; Wang et al., 2016), and multi-view feature selection/dimensionality reduction method (Yuan et al., 2021; Qiang et al., 2022). In the multi-view learning process, the collected multi-view dataset is apt to be high-dimensional, which is prone to dimension disasters. Hence, it is necessary to remove redundant features in multi-view data. Therefore, the multi-view feature selection has received wide attention.

The multi-view learning-based sparse optimization was proposed to jointly extract robust CSP features with *L*_2,1_-norm regularization, aiming to capture the shared salient information across multiple related spatial patterns. The method is termed as the multi-scale optimization (MSO) (Jiao et al., 2020). The MSO considers the optimization of the CSP feature set extracted from the spatial pattern and sub-band group and does not consider the influence of time window division on the multi-view learning model. The characteristics of CSP are affected by spatial pattern, frequency band, and time interval. On the basis of the MSO method, we consider time factors to optimize filter bands and time intervals over multiple channel sets within CSPs by multi-view learning. The novel framework is termed multi-domain feature joint optimization (MDFJO).

**Figure 3:**
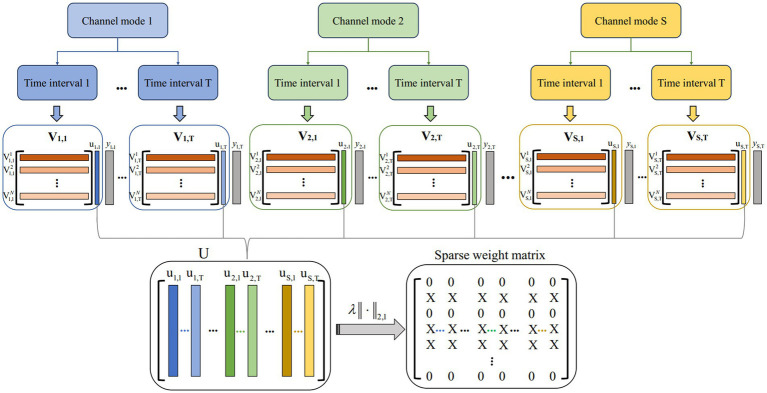
Multi-view model architecture based on *L*_2,1_. S and T represent the number of channel mode and time intervals, respectively. $V_{s,t}$ represents the CSP feature matrix in the *t*-th time interval over *s*-th channel mode. U is obtained from all views by a solving model. The sparse weight matrix is obtained after the matrix U is regularized by *L*_2,1_-norm regularization.

The multi-view model architecture based on *L*_2,1_ is shown in Figure 3. Suppose $V_{s,t}\in R^{N\times 2mk}$ represents the CSP feature matrix in the *t*-th time interval over *s*-th channel mode. The number of filters is *m* = 1, and *k* = 17 is the number of sub-bands. N is the total number of trials N = [N_1_; N_2_], where N_1_ is the total number of class 1 trials and N_2_ is the total number of class 2 trials. The channel mode is *s* = 1,2,…,S. The number of time intervals *t* = 1,2,…,T. The proposed multi-view learning model with *L*_2,1_-norm regularization is represented as follows:


(2) $$U=\underset{U}{\mathrm{argmin}}\frac{1}{2}\overset{S}{\underset{s=1}{\sum }}\overset{T}{\underset{t=1}{\sum }}||V_{s,t}u_{s,t}-y_{s,t}|{|}_{2}^{2}+\lambda ||U|{|}_{2,1}$$

where $\;u_{s,t}\in R^{2mk}\;$ represents the weight vector obtained in a single view with *s* and *t*. $U=\left[u_{1,1},\ldots {\mathrm{,u}}_{1,T}{\mathrm{,u}}_{2,1},\ldots {\mathrm{,u}}_{2,T}{\mathrm{,u}}_{S,1},\ldots {\mathrm{,u}}_{S,T}\right]$ is weight matrix obtained from all views. $y_{s,t}\in R^{N}$ is denoted as a class label with spatial pattern *s*-th and time window *t*-th. It should be noted that since each view in this study shares a common class label vector, the above convex optimization problem could be solved by the accelerated gradient descent method. The MATLAB toolkit “MALSAR” was developed to solve the above convex optimization problem and used the subfunction “mtleastR.m” to solve the above model (Zhou et al., 2011). ${\left\|U\right\|}_{2,1}$ is obtained by first computing the *L*_2_-norms of the rows $\;u_{s,t}$ and then the *L*_1_-norms of vector $\left(||\;u_{1}\;|\;{|}_{2},\;\;||\;u_{2}\;|{|}_{2},\;\;\ldots \;\;,\;\;||\;u_{T}\;|{|}_{2}\right).$ This encourages some rows of U to be zeros, thereby ensuring that CSP features corresponding to the non-zero rows will be selected across multiple views. Finally, the sparse weight matrix is obtained after the matrix U is regularized by *L*_2,1_-norm regularization.

#### 2.3.4 Feature sparsification strategy

**Figure 4:**
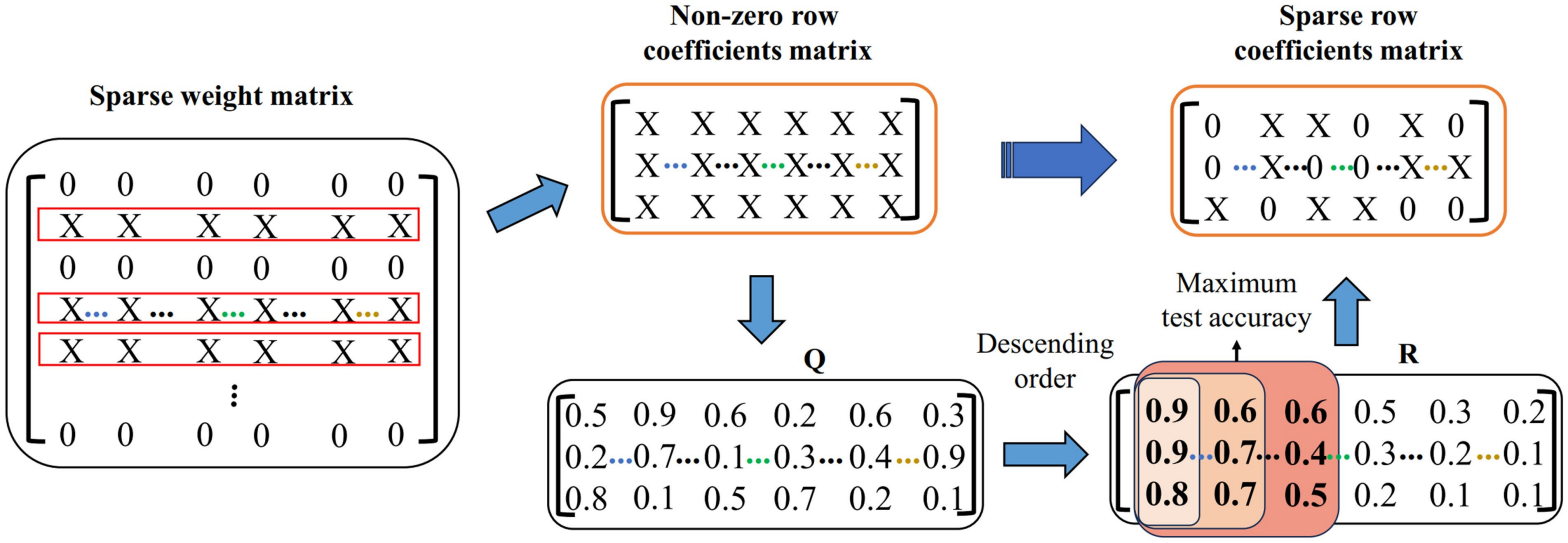
Feature sparsity strategy demonstration. Q represents the true value of X in a non-zero row coefficient matrix. R means by descending each row in Q.

We propose a feature sparsification strategy to further reduce the feature dimension. The demonstration of the feature sparsification strategy is shown in Figure 4. First, the non-zero row coefficients are extracted from the sparse weight matrix. Suppose that the matrix of all real values corresponding to X in the non-zero row coefficients is Q. Second, the weight vector of each row in Q is sorted according to the absolute value, forming a matrix R, and then it takes Ns times to calculate the test accuracy by extracting the corresponding feature set with column-by-column superposition. Finally, the corresponding features of the maximum test accuracy are calculated. For example, the features corresponding to the first three columns of coefficients (the red area coefficient in Figure 4) have the highest test accuracy. The sparse row coefficient matrix is obtained by keeping the coefficient corresponding to the maximum test accuracy and other coefficients set to 0.

The feature sparsification strategy will further sparsify the frequency band features on the time scale to reduce redundant information and computational cost. The most discriminative features are selected to improve the classification accuracy.

#### 2.3.5 Implementation steps of the MDFJO method

**Figure 5:**
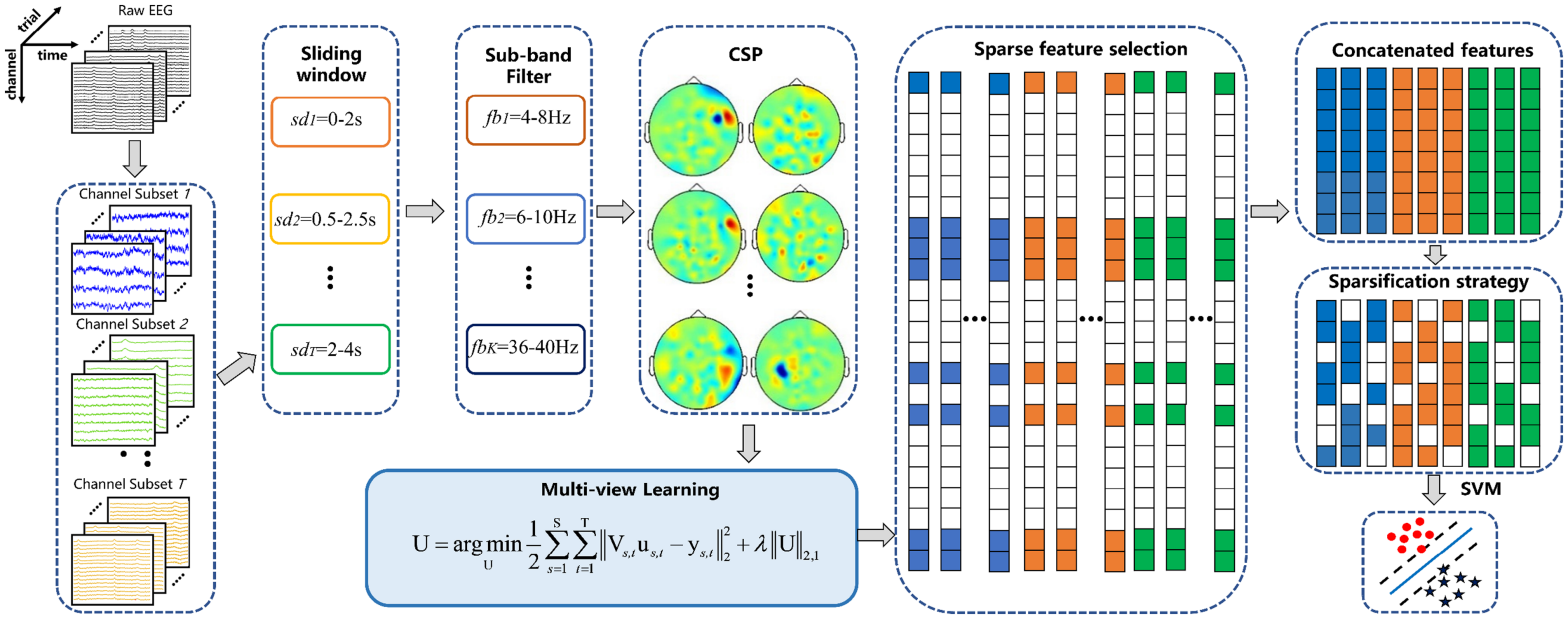
Framework diagram of the proposed MDFJO for the motor-imagery-related EEG classification. The method mainly includes channel pattern division, sub-band division, and time interval division, feature extracted by CSP and feature selection based on multi-view learning, feature sparsification strategy, and identification of test samples by SVM.

Figure 5 shows the overall block diagram of the proposed method. The proposed method can be described in detail as follows:

**Step (1)** The FDC was used for the preprocessing EEG data to rank the channel weights and divide channels into S channel combinations.
**Step (2)** According to the 5-fold cross-validation method, the EEG signals were divided into five parts, four of which were used as training samples, and the remaining one was used as test samples.
**Step (3)** For each channel combination mode, the CSP features of EEG signals in *k* sub-bands are calculated for each time window and the feature matrix $V_{s,t}\in R^{N\times 2mk}$ is obtained.
**Step (4)** The multi-view learning problem is solved by the accelerated gradient descent method to get U, and each non-zero row is sorted according to feature weight value.
**Step (5)** The optimal CSP feature set is obtained by the feature sparsification strategy.
**Step (6)** The features selected by all views are collected to train the SVM classifier and identify the class labels of the test samples.
**Step (7)** Repeat steps 3–6 until the 5-fold cross-validation is complete and output the average accuracy of the 5-fold test samples.

Algorithm 1 is the multi-domain feature joint optimization (MDFJO) based on multi-view learning for motor imagery EEG classification.

**Table:** 

**Algorithm 1**: MDFJO
**Input:** Original samples, the hyperparameter λ .
**Output:** 5-fold cross-validation test accuracy.
1: Channel pattern division based on FDC.
2: Divide the training samples and test samples.
3: Calculate CSP features in *k* sub-bands for each time window.
4: Form the feature matrix $V_{s,t}\in R^{N\times 2\mathrm{mk}}$ .
5: Solve the multi-view learning problem in (2) to get U.
6: Sort each non-zero row according to feature weight value.
7: Obtain the optimal CSP feature set by the feature sparsification strategy.
8: Train the SVM classifier by the features selected by all views and identify the test labels.
9: Repeat 3–8 until the 5-fold cross-validation is complete and output the average accuracy.

### 2.4 Comparative methods

The methods proposed in this article are compared with the following methods:

1. CSPs (s = 16, 32, and full channels): The CSP algorithm is used for feature extraction in three different channel modes of EEG, respectively. The frequency band was 4–40 Hz, and the time length was 0–4 s for Data 1 and 0–3.5 s for Data 2.
2. FBCSPs (s = 16, 32, and full channels): The time length is the same as CSP, and the sub-frequency bands are divided into 4–8 Hz, 6–10 Hz, 8–12 Hz…,36–40 Hz. CSP features are extracted for the whole-time interval in each sub-frequency band in different spatial patterns, and there is no time interval decomposition in this method. Then, the mutual information-based best individual feature (MIBIF) selection algorithm is used. CSP features of the frequency band are automatically selected. Based on the descending order arrangement of the mutual information value of the whole feature vector, the feature vectors corresponding to the first four sub-frequency bands are selected for subsequent training and testing.
3. SFBCSPs (s = 16, 32, and full channels): The time length and sub-band division are the same as FBCSP, and the Lasso is used for feature optimization in the fixed channel mode.
4. DFBCSP: The time and frequency band divisions are the same as FBCSP, and C3 is used to calculate Fisher’s score on each sub-band. Fisher’s score is sorted in descending order, and the corresponding features of the first four sub-bands are selected for subsequent training and testing.
5. MSO: The channel mode is consistent in Jiao et al. (2020). For the two datasets, the time is 0.5–2.5 s. The sub-band division is consistent with FBCSP. Multi-view learning is used to select the sub-band features in different spatial patterns.

The Wilcoxon signed-rank test is a non-parametric statistical test used to compare the difference between two correlated or paired samples, with the advantage that it does not require the assumption of a normal distribution of the data and is suitable for small samples and discontinuous data. Therefore, the Wilcoxon signed rank test is often used to calculate the differences between two EEG processing methods (Jin J. et al., 2020; Qi et al., 2021). Given the small sample size in this study, the statistical significance of each method versus MDFJO is assessed via the Wilcoxon signed-rank test.

It should be noted that the test results of the comparative methods in this article are based on the principles and parameters of the above methods and are not directly compared with the results of the literature.

### 2.5 Selection of hyperparameters

In the process of data analysis, several hyperparameters need to be determined. Among them, there are the regularization term sparsity γ in SFBCSP, the regularization term ρ of *L*_2,1_ norm in the multi-scale optimization method MSO. In this study, the regularization term λ of *L*_2,1_ norm and the number of featured Ns at the time level. In order to construct a better model, 5-fold cross-validation is used to determine the value of the hyperparameter. For each hyperparameter value, the corresponding training feature subsets are divided into five equal parts of which four copies are used to train the classification model, and the remaining one is used as a test set to evaluate the performance of the model. This process is repeated five times, and a 5-fold cross-validation average accuracy is obtained. The optimal value of the hyperparameter is determined for the highest average accuracy. The alternative set of hyperparameters is specified as follows: For SFBCSP, γ∈{0,0.01,…,1} . For the MSO, ρ∈{0,0.1,…,1} . For the MDFJO, λ∈{0,0.1,…,1} and $N_{s}\in \left\{1,2,\ldots \mathrm{,S}\times T\right\}$ .

## 3 Results

### 3.1 Classification performance

**Figure 6:**
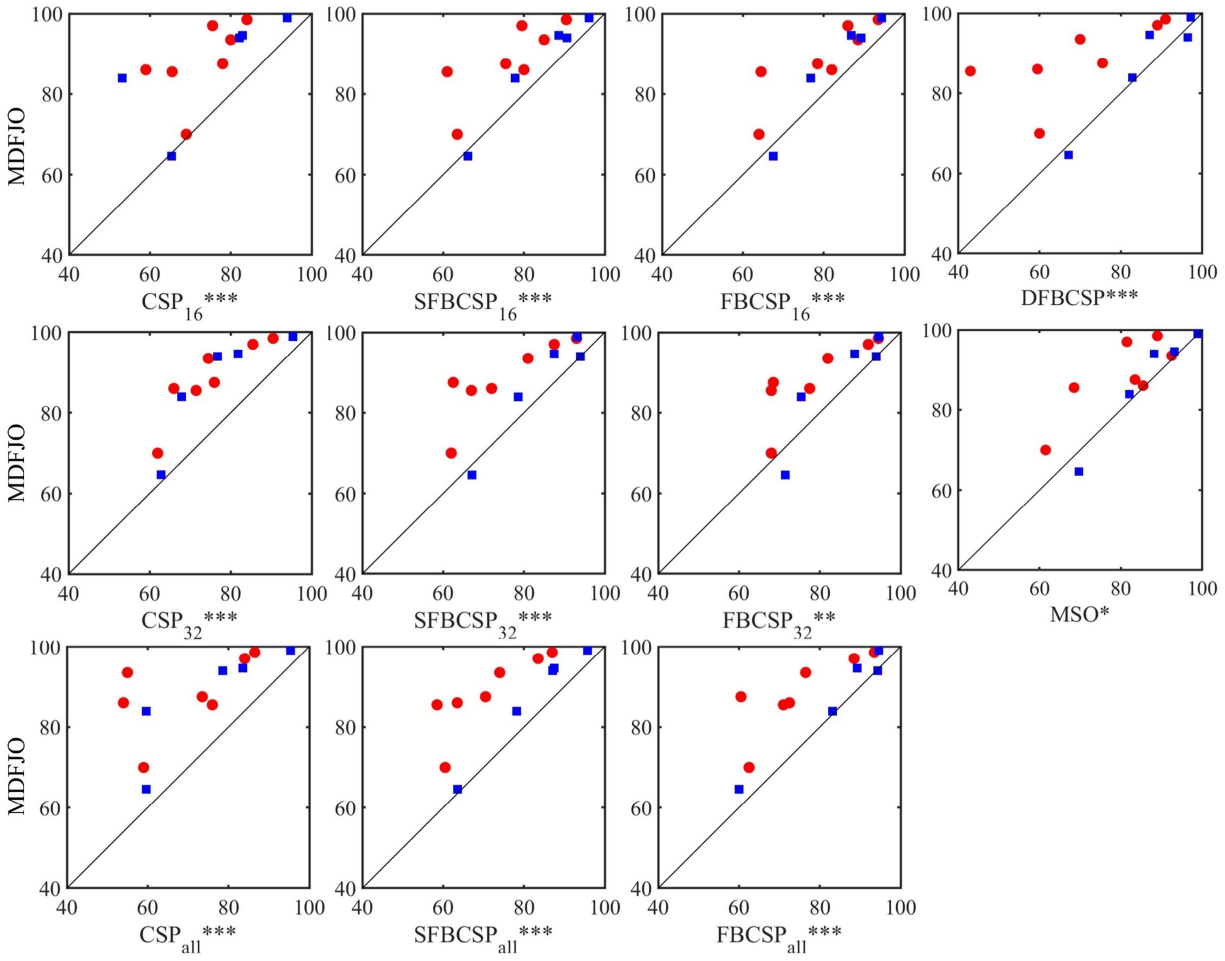
Test results of MDFJO and all comparison algorithms in all subjects (* *p* < 0.05, ** denotes *p* < 0.01, *** denotes *p* < 0.001). The red circle and the blue box represent the test results of all subjects in Data 1 and all subjects in Data 2, respectively.

The results of the proposed method MDFJO are compared with those of the traditional CSP, SFBCSP, FBCSP, DFBCSP, and MSO in different spatial patterns. Figure 6 shows the test results of MDFJO and all comparison methods in all subjects. The red circle and blue box represent the test results of all subjects in Data 1 and all subjects in Data 2, respectively. The results obtained by each method applied to the subjects are the average accuracy of 5-fold cross-validation. The Wilcoxon signed-rank test was used to analyze the statistical differences between MDFJO and each method. Finally, the classification result of MDFJO was significantly better than MSO (*p* < 0.05), FBCSP_32_ (*p* < 0.01), and other methods (*p* < 0.001). It is concluded that the classification performance of MDFJO is better than other methods.

Table 1 shows the average classification accuracy (%) of the proposed MDFJO and the existing methods on each dataset. As can be seen from Table 1, the average classification accuracy of the MDFJO in Data 1 and Data 2 is 88.29 and 87.21%, respectively. For Data 1 and data 2, the average classification accuracy of MDFJO is 87.75%, which was, respectively, higher than that of CSP_16_, CSP_32_, CSP_all_, SFBCSP_16_, SFBCSP_32_, SFBCSP_all_, FBCSP_16_, FBCSP_32_, FBCSP_all_, DFBCSP, and MSO improved by 13.50, 11.71, 15.21, 7.60, 8.21, 11.00, 6.50, 6.03, 8.10, 9.82, and 4.39%. These results indicate that the MDFJO could further improve the accuracy of the EEG classification in a motor imagery task.

**Table 1:** Average classification accuracy of MDFJO and existing methods on each dataset (%).

Methods	Data 1	Data 2	Mean
CSP_16_	73.00 ± 8.85	75.50 ± 16.11	74.25 ± 1.77
CSP_32_	75.14 ± 10.11	76.93 ± 12.68	76.04 ± 1.27
CSP_all_	69.71 ± 13.65	75.36 ± 15.59	72.54 ± 3.99
SFBCSP_16_	76.43 ± 10.79	83.86 ± 11.94	80.15 ± 5.25
SFBCSP_32_	75.00 ± 12.35	84.07 ± 11.28	79.54 ± 6.41
SFBCSP_all_	71.07 ± 11.14	82.43 ± 12.22	76.75 ± 8.03
FBCSP_16_	79.57 ± 11.49	82.93 ± 10.73	81.25 ± 2.38
FBCSP_32_	78.64 ± 11.34	84.79 ± 10.75	81.72 ± 4.34
FBCSP_all_	75.00 ± 12.35	84.29 ± 14.35	79.65 ± 6.57
DFBCSP	69.71 ± 17.18	86.14 ± 12.24	77.93 ± 11.62
MSO	80.29 ± 11.22	86.43 ± 11.24	83.36 ± 4.34
MDFJO	88.29 ± 9.62	87.21 ± 13.77	87.75 ± 0.76

Furthermore, it can be concluded from Table 1 that the average accuracy obtained by CSP_all_, SFBCSP_all_, and FBCSP_all_ are, respectively, lower than the results obtained by the 16–32 channel mode of the corresponding methods. Specifically, the average test accuracy of CSP_all_ (72.54%) was lower than that of CSP_16_ and CSP_32_ (1.71 and 3.50%). For SFBCSP_all_, the average test accuracy of 76.75% was lower than that of SFBCSP_16_ and SFBCSP_32_, which were 3.40 and 2.79%, respectively. For the method FBCSP_all_, the average test accuracy was 79.65%, which was lower than 1.60 and 2.07% for FBCSP_16_ and FBCSP_32_. The results show that channel selection of EEG can effectively improve classification accuracy.

In addition, the average test accuracy obtained by MDFJO on the two datasets is 4.39% higher than that obtained by MSO. One of the possible reasons is that MSO extracts sub-band features from the combined spatial pattern of multiple channels without using multiple time information. Therefore, the MDFJO can further improve the accuracy of motor imagery.

We performed a comparison of the classification performance of MDFJO with convolutional neural networks (CNN), as shown in Table 2. For Data 1 (BCI Competition IV dataset 1), the classification accuracy of our proposed MDFJO is 88.29 ± 9.62%, while the results of Yang et al. (2020) and Zhang et al. (2020) are 86.4 and 83.2 ± 3.5, respectively. For Data 2 (BCI Competition III dataset Iva), as can be seen from Table 2, the classification performance of the proposed method MDFJO is smaller than Miao et al. (2020) and Ortiz-Echeverri et al. (2019). A possible reason is that Data 2 has 118 channels, while Data 1 has 59 channels. Furthermore, Data 2 has 280 trials of the right hand and foot. The convolutional neural network (CNN) method is more suitable for data with a larger sample size and achieves better classification performance. However, the proposed MDFJO is more suitable for classification with a small number of trials for each subject.

**Table 2:** Comparison of classification accuracy for MDFJO and CNN on each dataset.

Study	Data	Deep learning modality	Strategy	Accuracy (%)
Yang et al. (2020)	Data 1 BCI-C IV-1	CNN	8-fold	86.4
Zhang et al. (2020)		CNN	10-fold	83.2 ± 3.5
MDFJO			5-fold	88.29 ± 9.62
Ortiz-Echeverri et al. (2019)	Data 2 BCI-C III-4a	CNN	10-fold	94.66
Miao et al. (2020)		CNN	10-fold	90
MDFJO			5-fold	87.21 ± 13.77

### 3.2 Combination of selected channels

**Figure 7:**
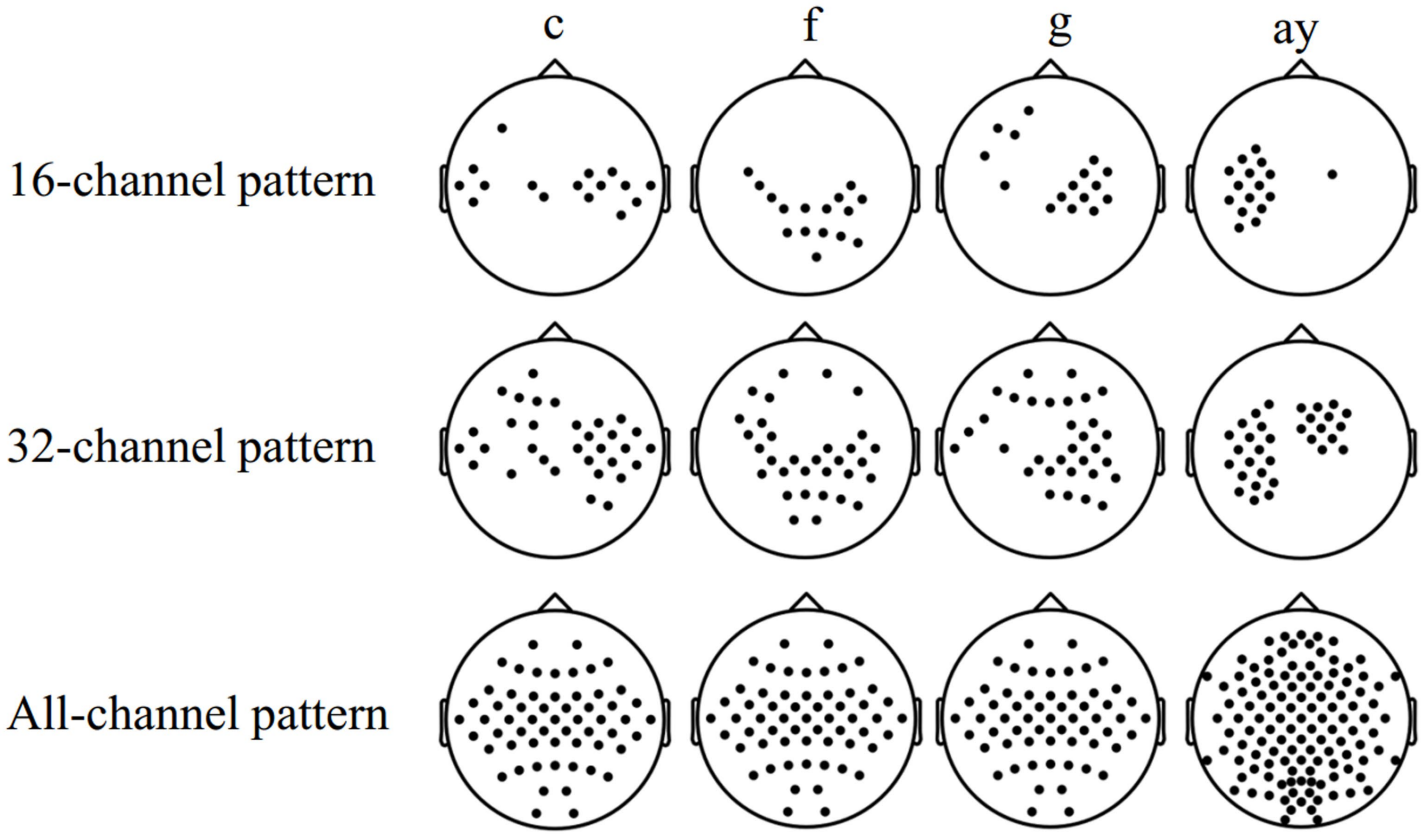
Three selected channel patterns obtained by using FDC, namely, the 16-32-all channel pattern mode.

Table 3 shows the comparison of the average classification accuracy (%) of subjects “c,” “f,” “g,” and “ay” in the MDFJO and the existing method. The 5-fold cross-validation accuracies of the MDFJO in subjects “c,” “f,” “g,” and “ay” are 85.50, 87.50, 93.50, and 94.64%, respectively. The three selected channel patterns obtained by using FDC are shown in Figure 7. For the subjects “c” and “g,” the motor imagery task involved the left and right hand. The chosen 16 and 32 channel patterns are also located in the central zone region of the sensorimotor cortex. Similarly, for subject “f,” the motor imagery task involved the left hand and right foot, and the 16 and 32 channel patterns are mainly distributed in the central and right-center regions of the sensorimotor cortex. For the subject “ay,” the imagery task was the imagination of the right hand and foot, and the two selected channel modes are mainly located in the left-sided region. In summary, the selected channels roughly matched the cortical activation regions generated by the corresponding motor imagery task.

**Table 3:** Comparison of average classification accuracy (%) of subjects c, f, g, and ay with the proposed method and the existing method.

Methods	C	f	G	ay
CSP_16_	65.50	78.00	80.00	82.86
CSP_32_	71.50	76.00	74.50	81.79
CSP_all_	76.00	73.50	55.00	83.57
SFBCSP_16_	61.50	75.50	85.00	88.57
SFBCSP_32_	67.00	62.50	81.00	87.50
SFBCSP_all_	58.50	70.50	74.00	87.50
FBCSP_16_	64.50	78.50	88.50	86.79
FBCSP_32_	68.00	68.50	82.00	88.57
FBCSP_all_	71.00	60.50	76.50	89.29
DFBCSP	43.00	75.50	70.00	87.14
MSO	68.50	83.50	92.50	93.21
MDFJO	85.50	87.50	93.50	94.64

### 3.3 Results of different channel combination modes

**Figure 8:**
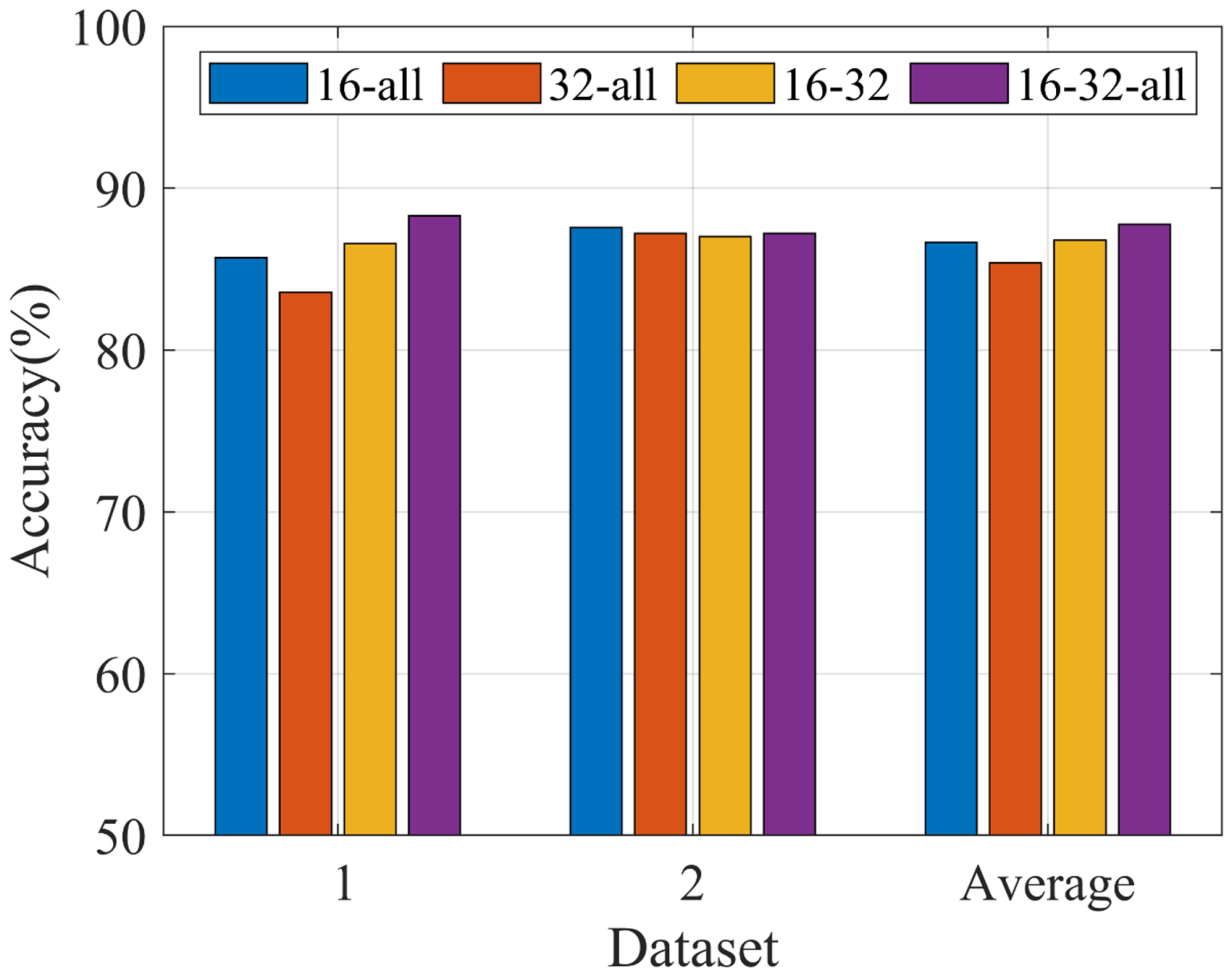
Classification accuracy comparison of MDFJO based on different combinations of channel patterns (16-all, 32-all, 16–32, and 16-32-all).

In order to study the classification performance of three different channel combination patterns based on MDFJO on two datasets, a 5-fold cross-validation is performed for each method, as shown in Figure 8. For Data 1, the average test accuracy of MDFJO based on channel mode 16-32-all is much higher than that of other channel mode methods. For Data 2, the MDFJO method of 16-32-all is slightly lower than the channel combination method of 16-all. Although the performance of each channel combination method is different, the average test accuracy of the MDFJO method based on the 16-32-all channel mode is larger than other combined channel modes.

## 4 Discussion

### 4.1 Parameter analysis

**Figure 9:**
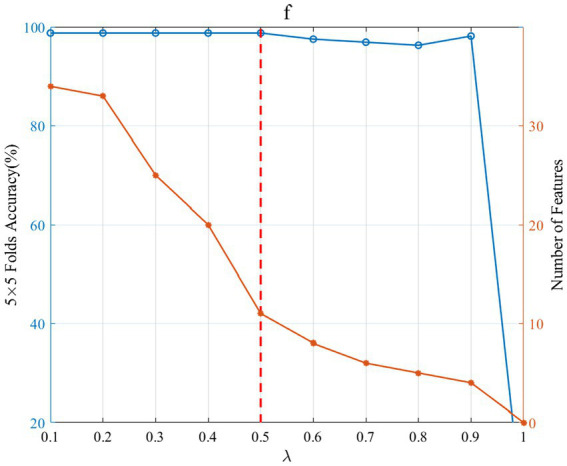
Effect of λ change on the selection number of sub-band features and the 5-fold cross-validation accuracy. The left blue ordinate is the 5 × 5 fold accuracy (%), and the right orange coordinate is the number of features. The blue curve and the orange curve, respectively, represent the change in 5 × 5 fold accuracy and the number of features. The red vertical dashed line indicates that when λ is 0.5, the accuracy is maximum and the number of features is small.

In the multi-view optimization model, the regularization coefficient λ has a great influence on the feature selection and the 5-fold cross-validation accuracy of the selected features. In this study, we investigated the feature group corresponding to the optimal λ so as to obtain the best test accuracy. Figure 9 shows the effect of λ change on the selection number of sub-band features and the 5-fold cross-validation accuracy. λ controls the number of selected features. Larger λ values result in fewer non-zero rows of the sparse matrix U in (2). A smaller λ value makes the sparse matrix U have more non-zero rows and thus more features. As can be seen from Figure 9, as λ grows, the number of selected sub-band features decreases, and when λ is 1, the number of selected features is 0. Thus, the larger λ value corresponding the higher classification accuracy should be selected as the optimal regular term λ . In this way, the sparsity of feature selection can be maintained without loss of accuracy. For subject “f,” the optimal λ value is 0.5, and the corresponding number of sub-band features is 11.

**Figure 10:**
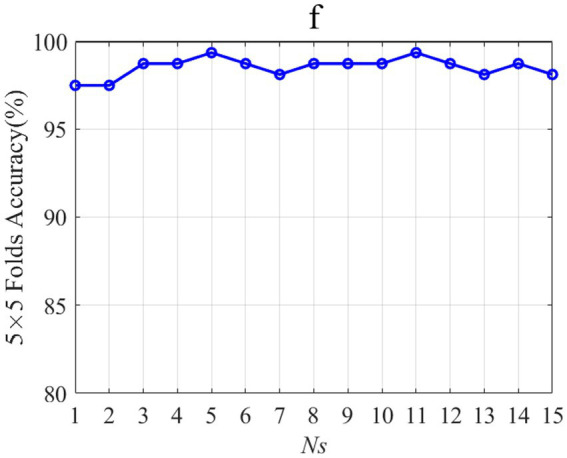
Influence of N_s_ on the accuracy of 5-fold cross-validation.

Figure 10 shows the influence of Ns change on the accuracy of 5-fold crossing validation accuracy. N_s_ is the number of weights on 15-time segments of the three-channel patterns. The feature corresponding to the smaller N_s_ with the highest average accuracy is considered the optimal feature. When N_s_ is 5 and 11, the 5-fold crossing validation accuracy is the highest. Considering that when N_s_ is 5, the selected features are the least, so N_s_ is 5 for subject “f.” It should be noted that subject “f” is only taken as an example to demonstrate the parameter selection method, and other subjects’ parameter selection methods are the same as subject “f.”

### 4.2 Analysis of corresponding features of sparse matrix

**Figure 11:**
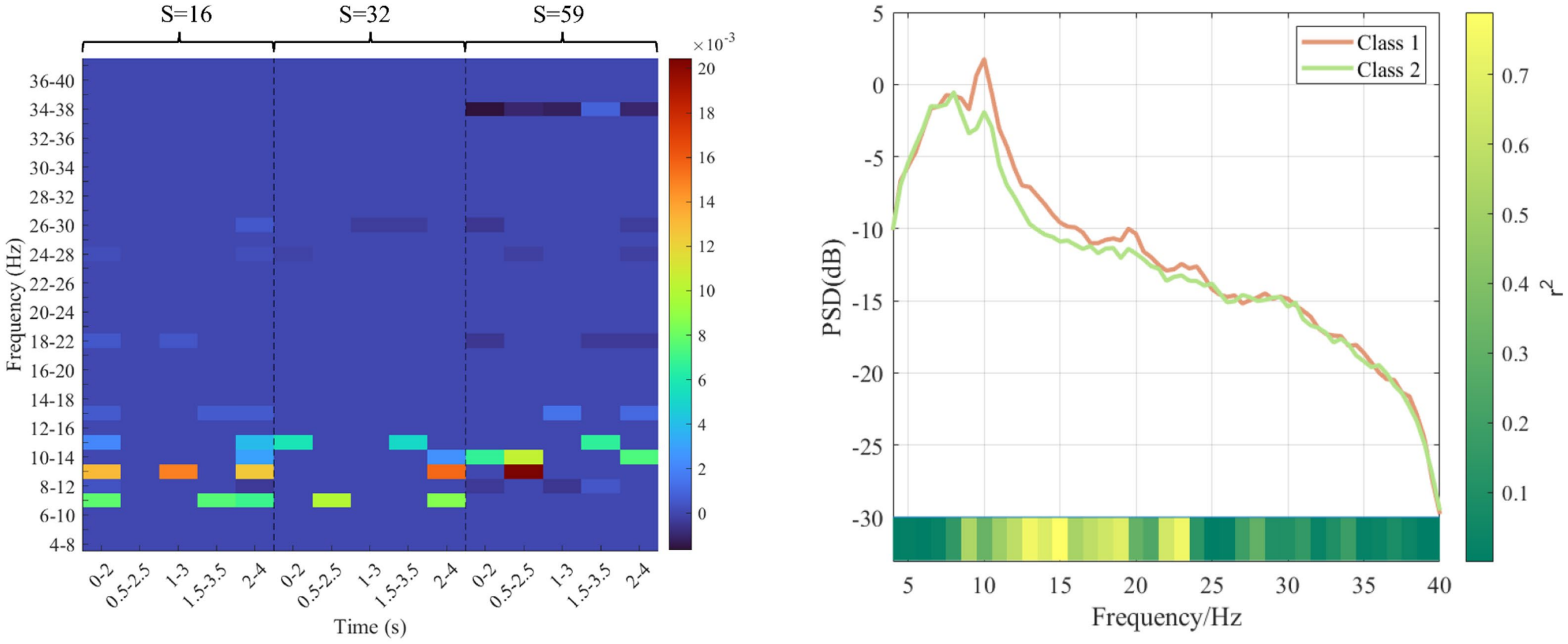
Sparse matrix and average power spectral density of subject f. The left subgraph is a sparse matrix. The darker red color represents a higher weight value. The right subgraph is average power spectral density. Orange and light green lines represent the average power spectral density curves of the two classes. The bottom color bar represents a *r*^2^ value.

As for subject “f,” the sparse matrix obtained based on MDFJO is shown in the left subgraph of Figure 11. The highest weight values are mainly concentrated at 6–16 Hz. To verify the effectiveness of the selected sub-band features, the average power spectral density is yielded for the two classes of data of 80 training trials, as shown in the right subgraph of Figure 11. Orange and light green lines represent the average power spectral density curves of the two classes. In this study, *r*^2^ is used to represent the difference between the two classes of power. A larger r^2^ indicates a larger difference between the two classes of power spectrum values. For two classes of power spectrum vectors *X*_1_ and *X*_2_. The r^2^ can be expressed as follows:


(3) $$r^{2}={\left(\frac{\sqrt{L_{1}L_{2}}}{L_{1}+L_{2}}\frac{mean\left(X_{1}\right)-mean\left(X_{2}\right)}{std\left(X_{1}\cup X_{2}\right)}\right)}^{2},$$

where *L*_1_ and *L*_2_ are, respectively, represented as the dimensions of two classes of power spectrum vectors. The color block at the bottom of the right subgraph of Figure 11 represents the size change of r^2^. It can be concluded that when the frequency is in the range of 8–20, there is a large difference in the spectral density of the two classes of power, which indirectly indicates the dilution validity of the selected sub-band features in the sparse matrix in Figure 11.

**Figure 12:**
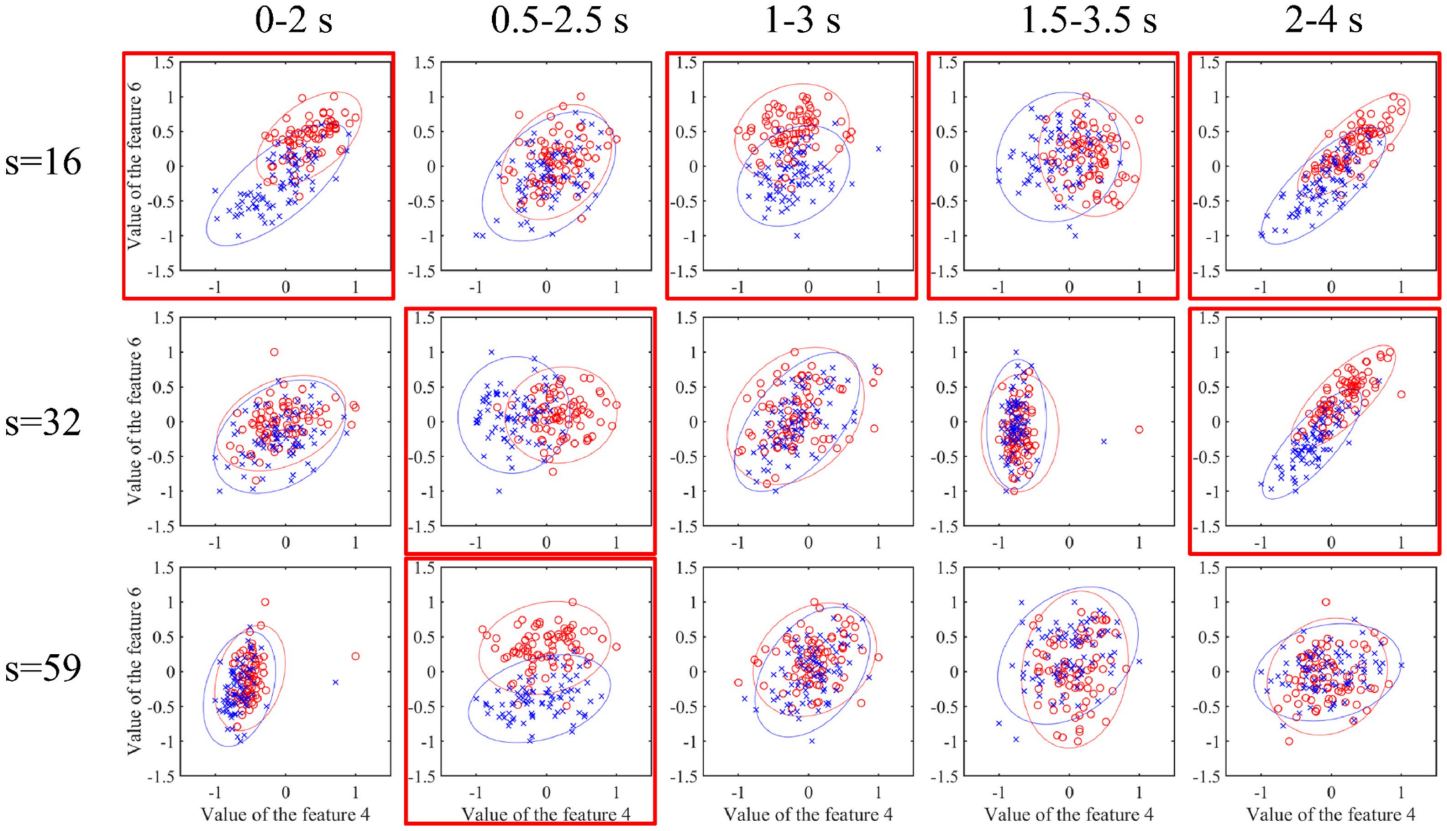
Characteristic difference of each channel mode at each time interval. The red-labeled subgraph indicates that the feature difference is greater than the other time intervals. The red circle and blue cross indicate two types of features.

At the same time, this work investigated the differences of the two classes of features corresponding to sub-band indexes 4 and 6 in each spatial pattern in each time interval, as shown in Figure 12. The red-labeled subgraph indicates that the feature difference is greater than other time intervals. The red circle and blue cross indicate two types of features. In addition, it can be seen that the high weight coefficient in the left subgraph of Figure 11 corresponds to the relatively large feature difference in Figure 12. This also shows the effectiveness of optimizing time interval features.

### 4.3 Limitations and future work

The proposed MDFJO is mainly suitable for EEG classification based on MI-based BCI. In future studies, we will extend this method to other event-related cortical potential decoding. In addition, only two datasets were used to verify the effectiveness of our method and more datasets will be used to test the effectiveness of this method in the future.

In our experimental study, we use an internal loop cross-validation step to determine the optimal hyperparameters of the proposed method. However, this optimal hyperparameter selection method is generally time-consuming, which would limit the application of the proposed method in BCI practice. The sparse Bayesian learning-based algorithm (Jin Z. et al., 2020) has been developed for automatic optimization of model hyperparameters. Therefore, in future studies, the sparse Bayes algorithm could be embedded into the proposed method to further improve the efficiency of hyperparameter selection.

## 5 Conclusion

In this study, the feature extraction method of the common spatial pattern is easy to be affected by time interval, bandpass filter, and channel selection. This results in weak feature differences and intention recognition accuracy. Therefore, the multi-domain feature joint optimization (MDFJO) based on the multi-view learning method was proposed. Compared with the CSP, SFBCSP, and FBCSP methods with 16-32-all channel mode as well as MSO, the MDFJO significantly improves the test accuracy. The feature sparsification strategy proposed in this article can effectively enhance classification accuracy. Future studies will investigate the performance of our proposed method on other types of BCI systems.

## Data availability statement

The original contributions presented in the study are included in the article/supplementary material, further inquiries can be directed to the corresponding authors.

## Author contributions

BS: Writing – original draft. ZY: Writing – review & editing. SY: Software, Writing – review & editing. JZ: Validation, Writing – review & editing. JW: Writing – review & editing.

## References

[ref1] Ang K. K. Chin Z. Y. Wang C. Guan C. Zhang H. (2012). Filter bank common spatial pattern algorithm on bci competition iv datasets 2a and 2b. Front. Neurosci. 6:39. doi: 10.3389/fnins.2012.00039 22479236 PMC3314883

[ref2] Ang K. K. Chin Z. Y. Zhang H. Guan C. (2008). “Filter bank common spatial pattern (FBCSP) in brain-computer interface” in 2008 IEEE International Joint Conference on Neural Networks (IEEE World Congress on Computational Intelligence) (Hong Kong: IEEE), 2390–2397.

[ref3] Baig M. Z. Aslam N. Shum H. P. Zhang L. (2017). Differential evolution algorithm as a tool for optimal feature subset selection in motor imagery EEG. Expert Syst. Appl. 90, 184–195. doi: 10.1016/j.eswa.2017.07.033

[ref4] Bekker A. J. Shalhon M. Greenspan H. Goldberger J. (2016). Multi-view probabilistic classification of breast microcalcifications. IEEE Trans. Med. Imaging 35, 645–653. doi: 10.1109/TMI.2015.2488019, PMID: 26452277

[ref5] Benjamin Blankertz F. L. Krauledat M. Dornhege G. Curio G. Müller K.-R. (2008). The Berlin brain-computer interface: accurate performance from first-session in BCI-naïve subjects. IEEE Trans. Biomed. Eng. 55, 2452–2462. doi: 10.1109/TBME.2008.923152, PMID: 18838371

[ref6] Blankertz B. (2008). Optimizing spatial filters for robust EEG single-trial analysis. IEEE Signal Process. Mag. 25, 41–56. doi: 10.1109/MSP.2008.4408441

[ref7] Blankertz B. Dornhege G. Krauledat M. Muller K. R. Curio G. (2007). The non-invasive Berlin Brain-Computer Interface: fast acquisition of effective performance in untrained subjects. NeuroImage 37, 539–550. doi: 10.1016/j.neuroimage.2007.01.051, PMID: 17475513

[ref8] Blankertz B. Muller K. R. Krusienski D. J. Schalk G. Wolpaw J. R. Schlogl A.. (2006). The BCI competition. III: Validating alternative approaches to actual BCI problems. IEEE Trans. Neural Syst. Rehabil. Eng. 14, 153–159. doi: 10.1109/TNSRE.2006.875642, PMID: 16792282

[ref9] Brusini L. Stival F. Setti F. Menegatti E. Menegaz G. Storti S. F. (2021). A systematic review on motor-imagery brain-connectivity-based computer interfaces. IEEE Trans. Human Machine Syst. 51, 725–733. doi: 10.1109/THMS.2021.3115094

[ref10] Chang C. C. Lin C. J. (2007). LIBSVM: A library for support vector machines. ACM Trans. Intell. Syst. Technol. 2, 1–27. doi: 10.1145/1961189.1961199

[ref11] Chepurova A. Hramov A. Kurkin S. (2022). Motor imagery: how to assess, improve its performance, and apply it for psychosis diagnostics. Diagnostics 12:949. doi: 10.3390/diagnostics12040949, PMID: 35453997 PMC9025310

[ref12] Choy C. S. Cloherty S. L. Pirogova E. Fang Q. (2023). Virtual reality assisted motor imagery for early post-stroke recovery: a review. IEEE Rev. Biomed. Eng. 16, 487–498. doi: 10.1109/RBME.2022.3165062, PMID: 35380970

[ref13] Faye I. Islam M. R. (2022). EEG channel selection techniques in motor imagery applications: a review and new perspectives. Bioengineering 9:726. doi: 10.3390/bioengineering9120726 36550932 PMC9774545

[ref14] Feng J. Yin E. Jin J. Saab R. Daly I. Wang X.. (2018). Towards correlation-based time window selection method for motor imagery BCIs. Neural Netw. 102, 87–95. doi: 10.1016/j.neunet.2018.02.011, PMID: 29558654

[ref15] Huang Y. Jin J. Xu R. Miao Y. Liu C. Cichocki A. (2021). Multi-view optimization of time-frequency common spatial patterns for brain-computer interfaces. J. Neurosci. Methods 365:109378. doi: 10.1016/j.jneumeth.2021.109378 34626685

[ref16] Jiao Y. Zhang Y. Chen X. Yin E. Jin J. Wang X.. (2019). Sparse group representation model for motor imagery EEG classification. IEEE J. Biomed. Health Inform. 23, 631–641. doi: 10.1109/JBHI.2018.2832538, PMID: 29994055

[ref17] Jiao Y. Zhou T. Yao L. Zhou G. Wang X. Zhang Y. (2020). Multi-view multi-scale optimization of feature representation for EEG classification improvement. IEEE Trans. Neural Syst. Rehabil. Eng. 28, 2589–2597. doi: 10.1109/TNSRE.2020.3040984, PMID: 33245696

[ref18] Jin J. Liu C. Daly I. Miao Y. Li S. Wang X.. (2020). Bispectrum-based channel selection for motor imagery based brain-computer interfacing. IEEE Trans. Neural Syst. Rehabil. Eng. 28, 2153–2163. doi: 10.1109/TNSRE.2020.3020975, PMID: 32870796

[ref19] Jin J. Miao Y. Daly I. Zuo C. Hu D. Cichocki A. (2019). Correlation-based channel selection and regularized feature optimization for MI-based BCI. Neural Netw. 118, 262–270. doi: 10.1016/j.neunet.2019.07.008 31326660

[ref20] Jin Z. Zhou G. Gao D. Zhang Y. (2020). EEG classification using sparse Bayesian extreme learning machine for brain–computer interface. Neural Comput. & Applic. 32, 6601–6609. doi: 10.1007/s00521-018-3735-3

[ref21] Laar B. V. D. Reuderink B. Bos P. O. Heylen D. (2010). Evaluating user experience of actual and imagined movement in BCI gaming. Int. J. Gaming Comput. Mediated Simulat. 2, 33–47. doi: 10.4018/jgcms.2010100103

[ref22] Li M. Ma J. Jia S. (2011). “Optimal combination of channels selection based on common spatial pattern algorithm” in 2011 IEEE International Conference on Mechatronics and Automation (Beijing: IEEE), 295–300.

[ref23] Li L. Xu G. Xie J. Li M. (2017). Classification of single-trial motor imagery EEG by complexity regularization. Neural Comput. Applic. 31, 1959–1965. doi: 10.1007/s00521-017-3174-6

[ref24] Maslova O. Komarova Y. Shusharina N. Kolsanov A. Zakharov A. Garina E.. (2023). Non-invasive EEG-based BCI spellers from the beginning to today: a mini-review. Front. Hum. Neurosci. 17:6648. doi: 10.3389/fnhum.2023.1216648, PMID: 37680264 PMC10480564

[ref25] Mcfarland D. J. Mccane L. M. David S. V. Wolpaw J. R. (1997). Spatial filter selection for EEG-based communication. Electroencephalogr. Clin. Neurophysiol. 103, 386–394. doi: 10.1016/S0013-4694(97)00022-2, PMID: 9305287

[ref26] Mcfarland D. J. Wolpaw J. R. (2017). EEG-based brain-computer interfaces. Curr. Opin. Biomed. Eng. 4, 194–200. doi: 10.1016/j.cobme.2017.11.004 29527584 PMC5839510

[ref27] Miao M. Hu W. Yin H. Zhang K. (2020). Spatial-frequency feature learning and classification of motor imagery EEG based on deep convolution neural network. Comput. Math. Methods Med. 2020, 1–13. doi: 10.1155/2020/1981728 PMC7387988 32765639

[ref28] Miao Y. Jin J. Daly I. Zuo C. Wang X. Cichocki A.. (2021). Learning common time-frequency-spatial patterns for motor imagery classification. IEEE Trans. Neural Syst. Rehabil. Eng. 29, 699–707. doi: 10.1109/TNSRE.2021.3071140, PMID: 33819158

[ref29] Miao M. Wang A. Liu F. (2017a). A spatial-frequency-temporal optimized feature sparse representation-based classification method for motor imagery EEG pattern recognition. Med. Biol. Eng. Comput. 55, 1589–1603. doi: 10.1007/s11517-017-1622-1, PMID: 28161876

[ref30] Miao M. Zeng H. Wang A. Zhao C. Liu F. (2017b). Discriminative spatial-frequency-temporal feature extraction and classification of motor imagery EEG: an sparse regression and weighted naive bayesian classifier-based approach. J. Neurosci. Methods 278, 13–24. doi: 10.1016/j.jneumeth.2016.12.010, PMID: 28012854

[ref31] Mohamed E. A. Yusoff M. Z. Malik A. S. Bahloul M. R. Adam D. M. Adam I. K. (2018). Comparison of EEG signal decomposition methods in classification of motor-imagery BCI. Multimed. Tools Appl. 77, 21305–21327. doi: 10.1007/s11042-017-5586-9

[ref32] Neuper C. Müller-Putz G. R. Scherer R. Pfurtscheller G. (2006). Motor imagery and EEG-based control of spelling devices and neuroprostheses. Prog. Brain Res. 159, 393–409. doi: 10.1016/S0079-6123(06)59025-9, PMID: 17071244

[ref33] Ortiz-Echeverri C. J. Salazar-Colores S. Rodríguez-Reséndiz J. Gómez-Loenzo R. A. (2019). A new approach for motor imagery classification based on sorted blind source separation, continuous wavelet transform, and convolutional neural network. Sensors 19:4541. doi: 10.3390/s19204541, PMID: 31635424 PMC6832153

[ref34] Padfield N. Zabalza J. Zhao H. Masero V. Ren J. (2019). EEG-Based Brain-Computer Interfaces Using Motor-Imagery: Techniques and Challenges. Sensors (Basel) 19:19. doi: 10.3390/s19061423 PMC6471241 30909489

[ref35] Pfurtscheller G. Brunner C. Schlogl A. Lopes Da Silva F. H. (2006). Mu rhythm (de)synchronization and EEG single-trial classification of different motor imagery tasks. NeuroImage 31, 153–159. doi: 10.1016/j.neuroimage.2005.12.003 16443377

[ref36] Pfurtscheller G. Neuper C. (2001). Motor imagery and direct brain-computer communication. Proc. IEEE 89, 1123–1134. doi: 10.1109/5.939829

[ref37] Qi F. Wu W. Yu Z. L. Gu Z. Wen Z. Yu T.. (2021). Spatiotemporal-filtering-based channel selection for single-trial EEG classification. IEEE Trans Cybern 51, 558–567. doi: 10.1109/TCYB.2019.2963709, PMID: 31985451

[ref38] Qiang L. A. Min M. A. Ly B. Ping Z. A. (2022). A supervised multi-view feature selection method based on locally sparse regularization and block computing. Inf. Sci. 582, 146–166. doi: 10.1016/j.ins.2021.09.009

[ref39] Quadrianto Novi C.G. Dat Tran Huy Xue Ping (2007). Sub-band common spatial pattern (SBCSP) for brain-computer interface. Proceedings of the 3rd International IEEE EMBS Conference on Neural Engineering. Kohala Coast, Hawaii, USA.

[ref40] Rodríguez-Bermúdez G. García-Laencina P. (2012). Automatic and adaptive classification of electroencephalographic signals for brain computer interfaces. J. Med. Syst. 36, 51–63. doi: 10.1007/s10916-012-9893-4, PMID: 23117792

[ref41] Sharma N. Sharma M. Singhal A. Vyas R. Malik H. Afthanorhan A.. (2023). Recent trends in EEG-based motor imagery signal analysis and recognition: a comprehensive review. IEEE Access 11, 80518–80542. doi: 10.1109/ACCESS.2023.3299497

[ref42] Tangermann M. Muller K. R. Aertsen A. Birbaumer N. Braun C. Brunner C.. (2012). Review of the BCI competition IV. Front. Neurosci. 6:55. doi: 10.3389/fnins.2012.00055 22811657 PMC3396284

[ref43] Thomas K. P. Guan C. Lau C. T. Vinod A. P. Ang K. K. (2009). A new discriminative common spatial pattern method for motor imagery brain-computer interfaces. I.E.E.E. Trans. Biomed. Eng. 56, 2730–2733. doi: 10.1109/TBME.2009.2026181 19605314

[ref44] Vidaurre C. Kramer N. Blankertz B. Schlogl A. (2009). Time domain parameters as a feature for EEG-based brain-computer interfaces. Neural Netw. 22, 1313–1319. doi: 10.1016/j.neunet.2009.07.020 19660908

[ref45] Wang J. Feng Z. Ren X. Lu N. Luo J. Sun L. (2020). Feature subset and time segment selection for the classification of EEG data based motor imagery. Biomed. Signal Process. Control 61:102026. doi: 10.1016/j.bspc.2020.102026

[ref46] Wang C. D. Lai J. H. Yu P. S. (2016). Multi-view clustering based on belief propagation. IEEE Trans. Knowl. Data Eng. 28, 1007–1021. doi: 10.1109/TKDE.2015.2503743

[ref47] Xu M. Chen Y. Wang D. Wang Y. Zhang L. Wei X. (2021). Multi-objective optimization approach for channel selection and cross-subject generalization in RSVP-based BCIs. J. Neural Eng. 18:046076. doi: 10.1088/1741-2552/ac0489, PMID: 34030144

[ref48] Xu C. Tao D. Xu C. (2013). A survey on multi-view learning. arXiv. doi: 10.48550/arXiv.1304.5634

[ref49] Yang J. Ma Z. Wang J. Fu Y. (2020). A novel deep learning scheme for motor imagery EEG decoding based on spatial representation fusion. IEEE Access 8, 202100–202110. doi: 10.1109/ACCESS.2020.3035347

[ref50] Yu J. Rui Y. Tang Y. Y. Tao D. (2014). High-order distance-based multiview stochastic learning in image classification. IEEE Trans. Cybernet. 44, 2431–2442. doi: 10.1109/TCYB.2014.2307862, PMID: 25415948

[ref51] Yuan H. Lo S. L. Yin M. Liang Y. (2021). Multi-view feature selection via sparse tensor regression. Int. J. Wavelets Multiresol. Informat. Process. 19:20. doi: 10.1142/S021969132150020X

[ref52] Zhang Y. Nam C. S. Zhou G. Jin J. Wang X. Cichocki A. (2019). Temporally constrained sparse group spatial patterns for motor imagery BCI. IEEE Trans. Cybern. 49, 3322–3332. doi: 10.1109/TCYB.2018.2841847, PMID: 29994667

[ref53] Zhang K. Xu G. Han Z. Ma K. Zhang S. (2020). Data augmentation for motor imagery signal classification based on a hybrid neural network. Sensors 20:4485. doi: 10.3390/s20164485, PMID: 32796607 PMC7474427

[ref54] Zhang Y. Zhou G. Jin J. Wang X. Cichocki A. (2015). Optimizing spatial patterns with sparse filter bands for motor-imagery based brain-computer interface. J. Neurosci. Methods 255, 85–91. doi: 10.1016/j.jneumeth.2015.08.004, PMID: 26277421

[ref55] Zhang Y. Zhou T. Wu W. Xie H. Zhu H. Zhou G.. (2021). Improving EEG decoding via clustering-based multi-task feature learning. IEEE Trans. Neural Netw. Learn. Syst. doi: 10.48550/arXiv.2012.06813 33556021

[ref56] Zhao X. Evans N. Dugelay J. L. (2014). A subspace co-training framework for multi-view clustering. Pattern Recogn. Lett. 41, 73–82. doi: 10.1016/j.patrec.2013.12.003

[ref57] Zhou J. Chen J. Ye J. (2011). “MALSAR: Multi-task learning via structural regularization.” Arizona State University. Available at: http://www.MALSAR.org.

[ref58] Zhou J. Meng M. Gao Y. Ma Y. Zhang Q. (2018). “Classification of motor imagery EEG using wavelet envelope analysis and LSTM networks” in 2018 Chinese Control And Decision Conference (CCDC): IEEE, 5600–5605.

